# Adaptive combination of Bayes factors as a powerful method for the joint analysis of rare and common variants

**DOI:** 10.1038/s41598-017-13177-7

**Published:** 2017-10-24

**Authors:** Wan-Yu Lin, Wei J. Chen, Chih-Min Liu, Hai-Gwo Hwu, Steven A. McCarroll, Stephen J. Glatt, Ming T. Tsuang

**Affiliations:** 1 Institute of Epidemiology and Preventive Medicine, College of Public Health, National Taiwan University, Taipei, Taiwan; 2 Department of Public Health, College of Public Health, National Taiwan University, Taipei, Taiwan; 3 Institute of Brain and Mind Sciences, College of Medicine, National Taiwan University, Taipei, Taiwan; 4 Genetic Epidemiology Core Laboratory, Division of Genomic Medicine, Research Center for Medical Excellence, National Taiwan University, Taipei, Taiwan; 5 Department of Psychiatry, College of Medicine and National Taiwan University Hospital, National Taiwan University, Taipei, Taiwan; 6 Stanley Center for Psychiatric Research, Broad Institute of MIT and Harvard, Cambridge, MA USA; 7 Program in Medical and Population Genetics, Broad Institute of MIT and Harvard, Cambridge, MA USA; 8 Department of Genetics, Harvard Medical School, Boston, MA USA; 9 Departments of Psychiatry and Behavioral Sciences and Neuroscience and Physiology, Medical Genetics Research Center, SUNY Upstate Medical University, Syracuse, New York USA; 10 Center for Behavioral Genomics, Department of Psychiatry, University of California San Diego, La Jolla, California USA; 11 Institute for Genomic Medicine, University of California San Diego, La Jolla, California USA

## Abstract

Multi-marker association tests can be more powerful than single-locus analyses because they aggregate the variant information within a gene/region. However, combining the association signals of multiple markers within a gene/region may cause noise due to the inclusion of neutral variants, which usually compromises the power of a test. To reduce noise, the “adaptive combination of *P*-values” (ADA) method removes variants with larger *P*-values. However, when both rare and common variants are considered, it is not optimal to truncate variants according to their *P*-values. An alternative summary measure, the Bayes factor (BF), is defined as the ratio of the probability of the data under the alternative hypothesis to that under the null hypothesis. The BF quantifies the “relative” evidence supporting the alternative hypothesis. Here, we propose an “adaptive combination of Bayes factors” (ADABF) method that can be directly applied to variants with a wide spectrum of minor allele frequencies. The simulations show that ADABF is more powerful than single-nucleotide polymorphism (SNP)-set kernel association tests and burden tests. We also analyzed 1,109 case-parent trios from the Schizophrenia Trio Genomic Research in Taiwan. Three genes on chromosome 19p13.2 were found to be associated with schizophrenia at the suggestive significance level of 5 × 10^−5^.

## Introduction

Multi-marker association tests can be more powerful than single-locus analyses because these tests combine variant information within a gene/region. Moreover, the multiple-testing penalty is moderate compared with that encountered in single-locus analyses. However, combining the association signals of multiple markers within a gene/region may cause noise due to the inclusion of neutral variants, which usually compromises the power of a multi-marker association test. To eliminate noise from neutral variants, the “adaptive combination of *P*-values” (ADA) method was proposed for the analyses of unrelated subjects^1,2^ and family data^3^.

The ADA method was originally proposed for rare-variant association testing^2^. While “rare” is frequently defined arbitrarily, here, according to Ionita-Laza *et al*.^4^, we defined variants with a minor allele frequency (MAF) $< 1/\sqrt{2n}$ as rare, where *n* is the number of individuals in the study. The per-site *P*-values were first calculated for each individual variant site, and the ADA method was used to truncate larger per-site *P*-values that were more likely to be attributed to neutral variants. The *P*-value is the probability of obtaining a statistic as extreme as or more extreme than the observed statistic under the null hypothesis (*H*_0_) of no association. However, a *P*-value provides no information regarding the alternative hypothesis (*H*_1_). For example, a *P*-value of 10^−9^ may appear to provide strong evidence against *H*_0_; however, if the test is low-powered, it may be almost as unlikely under *H*_1_ as under *H*_0_^5–7^. In genome-wide association studies (GWAS), the power to detect disease-associated single-nucleotide polymorphisms (SNPs) varies with MAFs. In this work, we show that truncating variants according to *P*-values is not optimal, when both rare and common variants are considered (see the subsection “Ranking by Bayes factor *vs*. *P*-value”).

Zhou and Wang^8^ have extended the ADA method to address both rare and common variants (namely, RC-ADA, or “rare and common variants by adaptive combination of *P*-values”). However, the RC-ADA method also truncates neutral variants according to their *P*-values. In RC-ADA^8^, rare variants and common variants are weighted according to *Beta*(*MAF*;1,25) and *Beta*(*MAF*;0.5,0.5)^4^, respectively, where *MAF* is the MAF of the considered SNP. Compared with the commonly used weight function *Beta*(*MAF*;1,25), *Beta*(*MAF*;0.5,0.5) decreases slowly as the MAF increases. RC-ADA preserves the associations of common variants by assigning them this weight function.

An alternative summary measure to the *P*-value is the Bayes factor (BF)^9,10^, which is the ratio of the probability of the data under the alternative hypothesis to that under the null hypothesis, as follows:

1 $$BF=\frac{\Pr (Data|{H}_{1})}{\Pr (Data|{H}_{0})},$$

where *H*_1_ and *H*_0_ are the alternative hypothesis and the null hypothesis, respectively. In this work, we show that truncating variants according to BFs is superior to truncating variants according to *P*-values, because BFs quantify the “relative” evidence supporting *H*_1_. Here, we propose an adaptive combination of BFs (ADABF) method by extending our previous ADA method^2^ and the “adaptive rank truncated product” (ARTP) method^11,12^. As described in the “Methods” section, the highest *k* BFs in favor of *H*_1_ are combined, in the observed sample and in each of the resamples, respectively. The optimal *k* that achieves the strongest signal is allowed to vary in the observed sample and in each of the resamples. Then, the significance of the gene/region is assessed by comparing the strongest signal in the observed sample with its counterparts in the resampling replicates.

The logic underlying this work can be traced back to the “variable-threshold (VT)” approach^13^. In the VT approach, Price *et al*. assume that a certain unknown MAF threshold, *T*, exists, and variants with MAFs lower than *T* are more likely to be disease-associated. Therefore, they compute the statistic for each MAF threshold and then search for the optimal MAF threshold with permutations. However, the MAF has little relevance to the association signals^14,15^. Disease-associated variants can be either rare or common. Here, we propose the ADABF method, which is based on the concept of VT, but we assume that a certain unknown BF threshold exists, and variants with BFs larger than this threshold are more likely to be disease-associated.

By performing extensive simulations with case-parent trios and unrelated case-control data, we find that our ADABF test is valid because the type I error rates match the nominal significance levels. Moreover, the ADABF test is more powerful than the other gene-based tests^16–19^. Various multi-marker methods and the single-locus transmission disequilibrium test (TDT)^20,21^ were then applied to the empirical data from the Schizophrenia Trio Genomic Research in Taiwan (S-TOGET)^22^.

## Results

### Simulation Results

**Figure 1:**
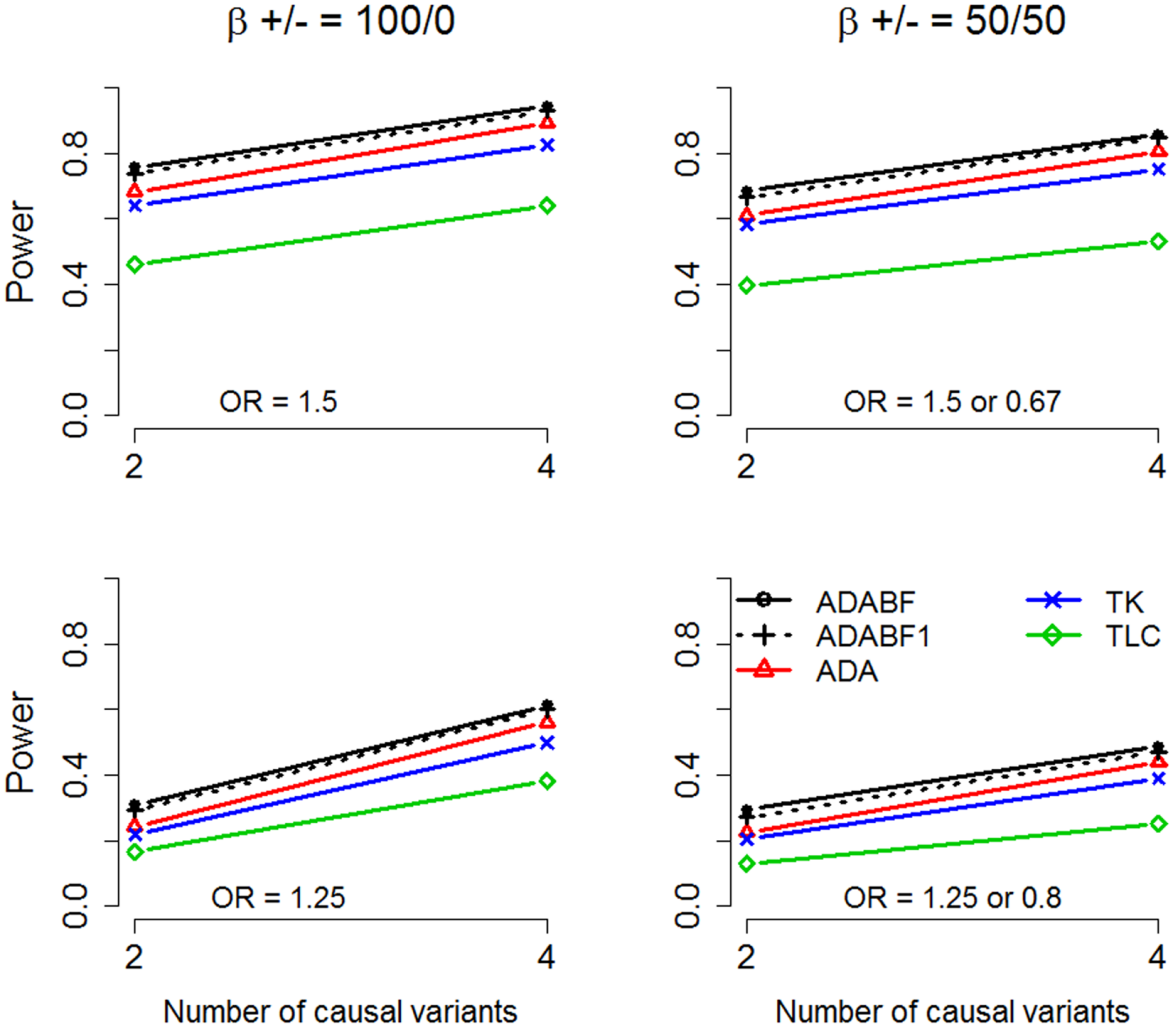
Simulation results of the case-parent trios. Top row: OR = 1.5 for a deleterious allele and OR = 0.67 for a protective allele; bottom row: OR = 1.25 for a deleterious allele and OR = 0.8 for a protective allele. Left column: all causal variants were deleterious; right column: ~50% of the causal variants were deleterious and the other ~50% were protective. The *x*-axis shows the number of causal variants, whereas the *y*-axis shows the power (given a significance level of 2.5 × 10^−6^).

Table 1 provides the type I error rates observed in 1,000,000 simulation replications (10,000 replications performed for each of the 100 Cosi data sets). All five tests are valid because their type I error rates match the nominal significance levels. Figures 1 (for case-parent trios) and 2 (for unrelated case-control data) present the power given the genome-wide significance level of 2.5 × 10^−6^ ($=0.05/20000$, corresponding to a Bonferroni correction for testing 20,000 independent genes^23,24^). The power for each scenario was evaluated using 10,000 simulation replicates (100 replicates for each Cosi data set). The ADABF method outperformed the other multi-marker tests because it excluded the variants with smaller BFs.

**Table 1:** Type I error rates in 1,000,000 simulation replications.

Significance level	ADABF	ADABF1	ADA	TK	TLC
2,000 case-parent trios					
*α* = 0.05	0.05012	0.05023	0.05041	0.05099	0.05062
*α* = 0.01	0.00919	0.00925	0.00908	0.01033	0.01016
*α* = 2.5 × 10^−6^	2 × 10^−6^	10^−6^	10^−6^	10^−6^	2 × 10^−6^
1,000 unrelated cases and 1,000 unrelated controls					
*α* = 0.05	0.04999	0.04999	0.04785	0.04983	0.05030
*α* = 0.01	0.00915	0.00919	0.00879	0.00994	0.01017
*α* = 2.5 × 10^−6^	2 × 10^−6^	3 × 10^−6^	2 × 10^−6^	10^−6^	2 × 10^−6^

As described in the “Methods” section, we created the “ADABF1” test representing our ADABF method coupled with another prior distribution. While the prior used in ADABF was chosen according to the WTCCC GWAS^6^, the purpose of adding ADABF1 was to evaluate the sensitivity of the results to the prior setting. ADABF1 (standard deviation of the prior distribution = 0.1) performed similarly to ADABF (standard deviation of the prior distribution = 0.2, following the WTCCC GWAS^6^). ADA also performed well because it truncated the variants with larger *P*-values. The popular kernel test (denoted by “TK”) was more powerful than the burden test (or linear combination test, denoted by “TLC”). Because the percentage of causal variants ($2/150$ or $4/150$, as described in “Simulation Study”) was not large, TK was generally more powerful than TLC. This result is consistent with the finding observed in rare-variant association testing for unrelated case-control data^25^.

Table 2 provides the average computation time (in seconds) for each test in our simulations, which was measured on a Linux platform with an Intel Xeon E5-2690 2.9 GHz processor and 8 GB memory. As described in the “Methods” section, we used the sequential resampling approach^26^ to compute the *P*-values of ADABF, ADABF1, and ADA. The minimum and maximum resampling numbers were set as 10^2^ and 10^7^, respectively. A longer time would be required to obtain a more significant result. Therefore, the average computation time increased as the power and the number of causal variants increased.

**Table 2:** Average computation time (in seconds) for each test in our simulations.

Number of causal variants	ADABF*	ADABF1*	ADA*	TK	TLC
2000 case-parent trios					
**0**	0.313	0.312	0.318	2.665	2.290
**2**	56.225	55.136	52.148		
**4**	89.127	88.149	85.243		
1,000 unrelated cases and 1,000 unrelated controls					
**0**	0.128	0.127	0.125	0.086	0.084
**2**	36.572	35.581	33.236		
**4**	64.145	64.132	63.259		

^*^We used the sequential resampling approach^26^ to compute the significance for ADABF, ADABF1, and ADA. The minimum and maximum numbers of resampling were set to be 10^2^ and 10^7^, respectively.

For unrelated case-control data, we also evaluated the “Variable Weight Test for testing the effect of an Optimally Weighted combination of variants” (VW-TOW)^18^. Because this test requires permutations to compute the *P*-values, we could not afford the computation time to evaluate it under the genome-wide significance level of 2.5 × 10^−6^ ($=0.05/20000$). Instead, we performed VW-TOW with 10,000 permutations and evaluated its power under the significance level of 0.01 (as shown in Figure S1). We found that its power performance was similar to that of TK. Zhou and Wang showed that ADA^2^ was more powerful than VW-TOW^18^ in testing the effects of both rare and common variants and rare variants alone^8^. Because ADABF is based on a similar concept in which the neutral variants are removed, it is not surprising that this method can outperform VW-TOW^18^.

When the haplotypes were generated according to the linkage disequilibrium (LD) patterns in Asians, the simulation results were similar to the abovementioned findings (Figures S2-S4 in our supplementary information).

### Application of Tests to the Genetic Analysis Workshop 17 Simulated Data

We further applied these multi-marker association tests to the Genetic Analysis Workshop 17 (GAW 17) simulated exome data^27^. Here, we analyzed two quantitative traits, i.e., Q4 and Q1. Q4 was not associated with any variants, whereas Q1 was influenced by 39 variants located in nine genes^27^. Conditional on the genotype data, the trait simulations were performed 200 times to generate 200 replicates for the 697 unrelated individuals.

TLC and TK were performed using the “SKAT” R package (version 1.2.1)^4,19,28^. The “Davies” method was used to compute the *P*-values^29^. VW-TOW was implemented using the R code downloaded from the authors’ website, i.e., http://www.math.mtu.edu/~shuzhang/software.html, and the number of permutations was set as 10^6^. The rare variant threshold (RVT) used in VW-TOW was set as $1/\sqrt{2n}$, where *n* is the sample size (697). Age and smoking status served as covariates adjusted in TLC, TK, and VW-TOW.

To perform ADABF, ADABF1, and ADA^2^, we first considered the linear regression for each locus as follows:

2 $$E(Y)={\beta }_{0}+{\beta }_{l}{G}_{l}+{\beta }_{A}Age+{\beta }_{S}Smoking,$$

where *Y* is the quantitative trait (Q4 or Q1) and *G*_*l*_ is the genotype score (0, 1, or 2) of the *l* ^th^ variant (*l* = 1, …, 24487). We obtained the maximum likelihood estimate (MLE) of *β*_*l*_ (*l* = 1, …, 24487) and the corresponding variance by fitting the linear regression (Eq. 2). The prior distribution of the true effect sizes (*β*_*l*_’s) was assumed to be *N*(0,*W*), where the prior variance was *W* = 0.2^2^ = 0.04 for ADABF and *W* = 0.1^2^ = 0.01 for ADABF1 (see Figure S5). The prior for ADABF was the prior setting from the WTCCC GWAS^6^, and was adopted for ADABF throughout this work. Although this prior was originally proposed for dichotomous traits^6^, we considered it suitable for standardized quantitative traits with a mean of 0 and a standard deviation of 1 (because this prior implied that 95% of the true effect sizes range from −0.4 to 0.4).

The *P*-values of ADABF, ADABF1, and ADA^2^ were all obtained using the sequential resampling approach^26^ in which the minimum and maximum numbers of resampling were set as 10^2^ and 10^7^, respectively.

To assess the type I error rates, for each replication, we sequentially tested the association of each gene with Q4. Summarizing 200 replications, we obtained 641,000 (= 200 × 3205) *P*-values for each multi-marker association method. Because Q4 did not depend on any variant, we assessed the type I error rates by calculating the percentages of the 641,000 *P*-values that were smaller than the significance level, i.e., $0.05/3205=1.56\times {10}^{-5}$, where 3,205 was the number of genes in the GAW 17 data. The first row in Table 3 provides the type I error rates. VW-TOW, ADABF, and ADABF1 yielded type I error rates that were the closest to the significance level ($0.05/3205=1.56\times {10}^{-5}$).

**Table 3:** Rejection rates when analyzing Q4 (no causal variants exist) and Q1 (causal variants exist) in the GAW 17 data

Trait	Analysis gene	Causal percentage^1^	No. of common causal variants (MAF and effect size)^2^	The mean effect size of causal variants^3^	Rejection rates^4^	ADABF	ADABF1	ADA	TK	TLC	VW-TOW
Q4	All 3205 genes	0	0	0	Type I error rates	1.7 × 10^−5^	1.7 × 10^−5^	1.9 × 10^−5^	1.0 × 10^−4^	1.1 × 10^−4^	1.2 × 10^−5^
Q1	*KDR*	$10/16=62.5 \%$	1 (MAF = 16.5%, *β* = 0.15)	$\bar{\beta }$ = 0.60	Power	0.915	0.845	0.955	0.525	0.965	0.940
	*FLT1*	$11/35=31.4 \%$	2 (MAF = 6.7%, *β* = 0.65); (MAF = 2.8%, *β* = 0.62)	$\bar{\beta }$ = 0.51		1.000	1.000	1.000	0.975	0.815	1.000
	*HIF1A*	$4/8=50 \%$	0	$\bar{\beta }$ = 0.26		0.130	0.130	0.080	0.180	0.090	0.000
	**Summation of the power for** ***KDR***, ***FLT1***, **and** ***HIF1A***					**2**.**045**	1.975	2.035	1.680	1.870	1.940

^1^Causal percentage = #(causal variants)/#(total variants).

^2^Following Ionita-Laza *et al*.^4^, here, we define variants with MAF $\ge 1/\sqrt{2n}=1/\sqrt{2\times 697}=2.678 \%$ as common, where *n = *697 is the sample size in the GAW 17 data. The effect size, *β*, is the displacement in mean levels of Q1 for each copy of the minor allele^27^.

^3^ $\bar{\beta }$ is the arithmetic mean of the *β*’s for the causal variants in the gene.

^4^The rejection rates given the significance level = $0.05/3205=1.56\times {10}^{-5}$, where 3205 is the number of genes in the GAW 17 data set. When analyzing Q4, in which no causal variants were simulated, the rejection rates were type I error rates. When analyzing Q1, which was influenced by certain causal variants, the rejection rates represented power.

To quantify the power, we analyzed the association of all the nine causal genes that influenced Q1^27^. Among the nine genes, the power for six genes (*ARNT*, *ELAVL4*, *FLT4*, *HIF3A*, *VEGFA*, and *VEGFC*) was smaller than 0.1 for all the tests and it was impossible to compare the different methods using this very low power. The second to fourth rows shown in Table 3 provide the power for the remaining three causal genes, i.e., *KDR*, *FLT* *1*, and *HIF1A*, respectively. For each gene and each method, we obtained 200 *P*-values after analyzing all the 200 replicates. We quantified the power by calculating the percentage of the 200 *P*-values that were smaller than the significance level ($0.05/3205=1.56\times {10}^{-5}$, where 3,205 was the number of genes in the GAW 17 exome data).

Overall, ADABF was the most powerful test. It provided the largest summation of power for detecting the three genes. Different from the above simulation results, TLC was not the least powerful test for the following two reasons:

1. According to the simulation model of the GAW 17 data, for all causal variants, the minor allele was associated with a higher mean Q1^27^. Therefore, the power of TLC would not be compromised due to the coexistence of trait-increasing and trait-decreasing variants.
2. As described in the above simulation results, TLC is vulnerable to a small causal percentage (i.e., the percentage of causal variants among all variants in the gene). In contrast to the small causal percentage in the abovementioned simulations ($2/150$ or $4/150$), the causal percentages of the three genes were all larger than 30% here (shown in Table 3).

### Application to the Schizophrenia Trio Genomic Research in Taiwan (S-TOGET)

Schizophrenia is a highly heritable disease^30^. Previous studies have suggested that 1/3 to 1/2 of the genetic variants responsible for schizophrenia are common^31,32^, and these variants are genotyped using GWAS arrays. Therefore, GWAS is an important tool for exploring the genetic architecture of schizophrenia.

A portion of the Taiwanese case-parent trios obtained from the S-TOGET from 2009 to 2014 were subjected to GWAS genotyping^22^, approved by the Research Ethics Committee of the National Taiwan University Hospital (NTUH-REC no. 200810016 R). We confirmed that all experiments were performed in accordance with the relevant guidelines and regulations.

Totally 3,374 subjects were genotyped using the PsychChip array, which was developed by the Psychiatric Genomics Consortium (PGC) and Illumina (Illumina, San Diego, CA). After removing individuals with call rates < 98%, Mendelian errors, or sex inconsistency, 1,109 case-parent trios were used for analysis.

The PsychChip array (PsychChip_15048346_B) included ~580,000 markers in total. After removing invariant markers, markers with call rates < 98%, and markers that were significant for the Hardy-Weinberg equilibrium test (*P*-value < 10^−6^ in controls), 325,994 autosomal markers were retained for analysis.

The PsychChip array is a genotyping chip customized for psychiatric phenotypes. Unlike most commercial GWAS arrays, the PsychChip array allows investigators to simultaneously examine multiple genetic variants, including SNPs and rare variants. Of the 325,994 autosomal variants, 65,658 variants had MAFs < 1%, and 21,989 variants had MAFs ranging from 1% to 5%, where the MAFs were calculated according to the parents of the 1,109 trios.

We first used the single-variant TDT^20,21^ to analyze the 1,109 case-parent trios. As shown in the bottom-right plot of Fig. 3, no variant was found to be associated with schizophrenia at the genome-wide significance level of 5 × 10^−8^ ($0.05/1,000,000$) or at the suggestive significance level of 10^−6^ ($1/1,000,000$)^33,34^.

**Figure 2:**
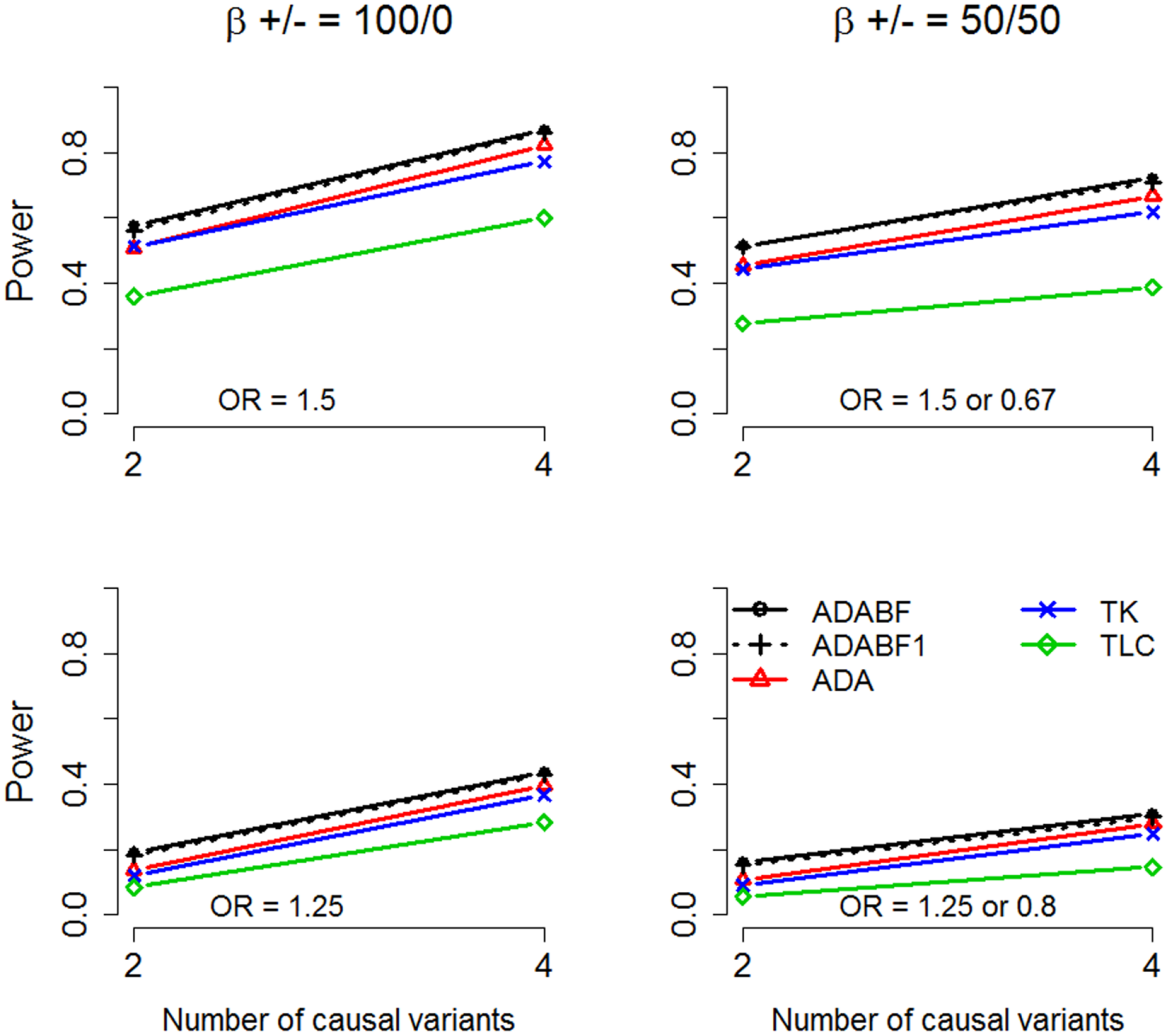
Simulation results of the unrelated cases and controls. Top row: OR = 1.5 for a deleterious allele and OR = 0.67 for a protective allele; bottom row: OR = 1.25 for a deleterious allele and OR = 0.8 for a protective allele. Left column: all causal variants were deleterious; right column: ~50% of the causal variants were deleterious and the other ~50% were protective. The *x*-axis shows the number of causal variants, whereas the *y*-axis shows the power (given a significance level of 2.5 × 10^−6^).

**Figure 3:**
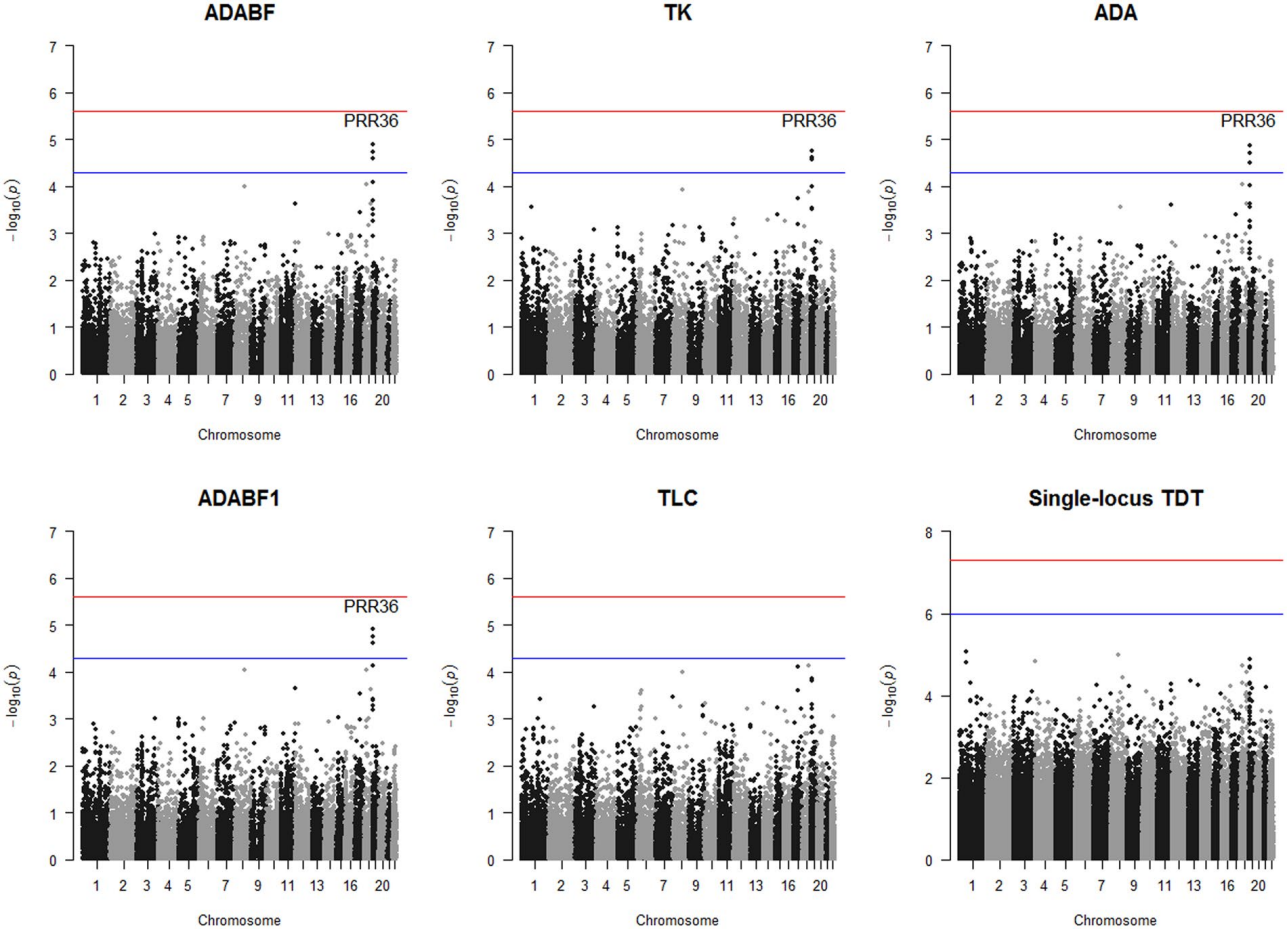
Manhattan plots of the Schizophrenia Trio Genomic Research in Taiwan (S-TOGET) data. Red lines indicate the genome-wide significance levels, i.e., 2.5 × 10^−6^ for the gene-based analyses and 5 × 10^−8^ for the single-locus analysis, respectively. Blue lines mark the suggestive significance levels, i.e., 5 × 10^−5^ for the gene-based analyses and 10^−6^ for the single-locus analysis, respectively. The three points surpassing the suggestive significance threshold represent the signals of the three genes (*EVI5L*, *PRR36*, and *LYPLA2P2*), although only the most significant gene (*PRR36*) is labeled.

We then resorted to multi-marker analyses. Because the SNP positions of the S-TOGET data were based on the human genome GRCh37/hg19 assembly, we mapped variants into genes according to the same assembly in the UCSC Genome Bioinformatics database (http://www.genome.ucsc.edu). We also included the 5’ and 3′ flanking regions of each gene. The 5′ flanking region may contain regulatory sequences such as promoters that control gene transcription. The 3′ flanking region may contain sequences that terminate transcription. Multi-marker analyses may include ±5 kb^35^, ±10 kb^36^, ±20 kb^37^, or ±30 kb^38^ flanking regions of a gene. Because incorporating additional flanking sequences increases the coverage of more distant regulatory elements, we grouped the variants within ± 30 kb flanking regions of a gene into a multi-marker analysis according to Song *et al*.^38^. In total, there were 24,769 autosomal genes.

TLC and TK were performed using the “rvTDT” R package (version 1.0)^16^. ADABF, ADABF1, and ADA^2^ were performed using the sequential resampling approach^26^, in which the minimum and maximum numbers of resampling were set as 10^2^ and 10^7^, respectively. The genome-wide significance level for the gene-based analyses is usually determined at 2.5 × 10^−6^ ($0.05/20,000$)^23,24^, and the suggestive significance level is set at 5 × 10^−5^ ($1/20,000$), respectively.

As shown in Fig. 3 and Table 4, no gene was found to be associated with schizophrenia at the genome-wide significance level of 2.5 × 10^−6^. Three genes on chromosome 19p13.2, including *EVI5L* (ecotropic viral integration site 5 like), *PRR36* (proline rich 36), and *LYPLA*2*P2* (lysophospholipase II pseudogene 2), were detected to be associated with schizophrenia at the suggestive significance level of 5 × 10^−5^. This is a consistent result across all the five gene-based association tests except TLC.

**Table 4:** Three genes on chromosome 19p13.2 detected to be associated with schizophrenia at the suggestive significance level of 5 ×10^−5^.

Gene	Chr.	Analysis region^1^ (Base pairs)	#(variants)	*P*-value				
				ADABF^2^	ADABF1^2^	ADA^2^	TK	TLC
*EVI5L*	19	7865161–7959862	11	1.82 × 10^−5^	1.77 × 10^−5^	1.95 × 10^−5^	2.40 × 10^−5^	0.05741
*PRR36*		7903605–7969326	8	1.27 × 10^−5^	1.21 × 10^−5^	1.35 × 10^−5^	1.71 × 10^−5^	0.01646
*LYPLA2P2*		7913504–7975117	8	2.61 × 10^−5^	2.39 × 10^−5^	3.14 × 10^−5^	2.70 × 10^−5^	0.00015

^1^The analysis regions were based on the human GRCh37/hg19 assembly. Following Song *et al*.^38^, we also grouped the variants within ± 30 kb flanking regions of a gene into a multi-marker analysis.

^2^The *P*-values of ADABF, ADABF1, and ADA were obtained with 10^7^ resampling replicates.

The 13 SNPs in the *EVI5L*-*PRR36*-*LYPLA2P2* region are described in Table 5. Some of the odds ratios (ORs) of the minor alleles compared with the major alleles were greater than 1, whereas others were less than 1. The TLC test could suffer from a power loss in this situation. Hence, it was not surprising that TLC could not identify the association signal of this region.

**Table 5:** The 13 SNPs in the *EVI5L- PRR36- LYPLA2P2* region.

**SNP** ^1^	**Position**	**Included in the analysis for**			**Minor allele**	**Major allele**	**MAF** ^2^	***b***	***c***	**Odds ratio** ^3^	**Chi-square statistic**	***P*** **-value of single-locus TDT**	**Bayes factor** ^4^
		***EVI5L***	***PRR36***	***LYPLA2P2***									
rs12980113	7868715	V			T	C	0.442	583	519	1.123	3.72	0.05386	1.58
rs580984	7881030	V			G	A	0.483	607	484	1.254	13.87	0.000196	161.52
rs4804827	7898541	V			T	C	0.032	67	73	0.918	0.26	0.6121	0.70
**rs652260**	**7900562**	**V**			**C**	**T**	**0**.**467**	**620**	**485**	**1**.**278**	**16**.**49**	**4**.**88** × 10^−5^	**532**.**33**
**rs1651016**	**7904297**	**V**	**V**		**A**	**G**	**0**.**435**	**620**	**479**	**1**.**294**	**18**.**09**	**2**.**11** × 10^−5^	**1095**.**42**
**rs555609**	**7913974**	**V**	**V**	**V**	**T**	**C**	**0**.**450**	**617**	**483**	**1**.**277**	**16**.**32**	**5**.**34** × 10^−5^	**492**.**48**
rs537188	7921623	V	V	V	A	G	0.102	203	215	0.944	0.34	0.5572	0.51
rs747990	7931525	V	V	V	A	G	0.430	481	604	0.796	13.94	0.000188	167.14
**rs525420**	**7936208**	**V**	**V**	**V**	**G**	**A**	**0**.**426**	**474**	**618**	**0**.**767**	**18**.**99**	**1**.**32** × 10^−5^	**1641**.**32**
rs483808	7957481	V	V	V	C	T	0.419	514	551	0.933	1.29	0.2569	0.53
rs533822	7959480	V	V	V	G	A	0.450	552	525	1.051	0.68	0.4107	0.40
exm1417450	7963948		V	V	A	G	0.097	208	181	1.149	1.87	0.171	0.95
rs4804833	7970635			V	A	G	0.411	496	526	0.943	0.88	0.348	0.45

^1^The analysis for the *EVI5L* gene contained 11 variants spanning from 7865161 to 7959862 base pair (bp), and the four SNPs shown in bold type were prioritized by ADABF, ADABF1, and ADA. The analysis for the *PRR36* gene included 8 variants from 7903605 to 7969326 bp, and rs1651016, rs555609, and rs525420 were prioritized. The analysis for the *LYPLA2P2* gene contained 8 variants from 7913504 to 7975117 bp, and rs555609 and rs525420 were prioritized.

^2^The minor allele frequencies (MAFs) were calculated according to the founder genotypes.

^3^The odds ratio of the minor allele compared with the major allele, *b*/*c*, where *b* is the number of transmissions of the minor allele from heterozygous parents to affected offspring, and *c* is the number of transmissions of the major allele.

^4^The prior distribution of log(ORs) was assumed to be a normal distribution with a mean of 0 and a standard deviation of 0.2.

## Discussion

In this work, we proposed the “adaptive combination of Bayes factors” (ADABF) method, which is applicable to a mixture of common and rare variants and can be applied to GWAS or next-generation sequencing (NGS) data.

Chromosome 19p13.2 has been found to be associated with panic disorder^39^. Based on our analysis for the S-TOGET trio data, three genes in this region, including *EVI5L*, *PRR36*, and *LYPLA2P2*, were detected to be associated with schizophrenia at the suggestive significance level of 5 × 10^−5^. Four multi-marker tests including ADABF, ADABF1, ADA, and TK all suggest that *PRR36* is the most significant gene. This gene encodes a large protein - Proline Rich Protein 36 (PRP36)^40^. The second significant gene identified by the four multi-marker tests is *EVI5L*. It is also a protein-coding gene, but its function remains unknown^41^. The third significant gene identified by the four tests is *LYPLA2P2*, which is a pseudo gene^42^.

The gene next to the *EVI5L*-*PRR36*-*LYPLA2P2* region (7865161–7975117 base pair) is *MAP2K7* (mitogen-activated protein kinase kinase 7, also known as the “*MKK7*” gene, 7968665–7979363 base pair). Knocking out *MAP2K7* results in schizophrenia-like behavioral deficits in mice^43–45^. A substantial effect size was observed for common variants in a case-control sample from the Glasgow area and a replication sample of Northern European descent^46,47^.

In our analysis of the *EVI5L* gene, the most prominent signal was achieved by combining the top four significant SNPs (see Table 5, i.e., rs525420, rs1651016, rs652260, and rs555609). This was a consistent prioritization of SNPs across ADABF, ADABF1, and ADA. These four SNPs have not been reported to be associated with schizophrenia. As shown in these four SNPs, the ORs of the minor alleles compared with the major alleles are larger than 1.25 or smaller than 0.8, corresponding to one of our simulation scenarios. To detect variants with smaller effect sizes, the number of case-parent trios must be increased.

In this work, we used the prior in the WTCCC GWAS^6^ [*β*~*N*(0,*W*), with a variance of *W* = 0.2^2^ = 0.04] as the prior for ADABF. To evaluate the sensitivity of our results to this choice, we also considered another prior variance, i.e., *W* = 0.1^2^ = 0.01. We found that our simulation and the S-TOGET results were very stable across these two settings. As noted by Stephens and Balding^5^, *W* can be chosen dependently on the MAF according to prior settings that are believed to best fit the underlying genetic architecture of a disease. Therefore, theoretically, we can develop better ways to prioritize SNPs.

With the advent of NGS technology, there has been a great interest in rare-variant association testing. However, both rare and common variants contribute to the etiology of complex diseases such as the Hirschsprung disease^48^, schizophrenia^49^, and type 2 diabetes^50^. Certain specialized arrays such as PsychChip were designed for the detection of both common and rare variants. There is a need to develop a powerful method for the joint analysis of rare and common variants. Compared with ADA^2^ and RC-ADA^8^, our ADABF method is recommended for its applicability to variants with a wide spectrum of MAFs. Compared with other multi-marker association tests such as TLC^16,51^, TK^4,16,19,28^, and VW-TOW^18^, our ADABF method is recommended for its robustness to the inclusion of neutral variants.

## Methods

Here, we describe the method to analyze case-parent trios, but it can be generalized to unrelated case-control analyses. For a variant with two alleles, i.e., *M*_1_ (the allele of interest) and *M*_2_, the TDT tests whether the *M*_1_ allele is transmitted to an affected child more often than the *M*_2_ allele from heterozygous parents^20^.

Let OR be the odds ratio of allele *M*_1_ compared with allele *M*_2_. We denote $\hat{\beta }$ as the MLE of log(OR). According to the asymptotic normality of MLE, $\hat{\beta } \sim N(\beta ,V)$. Let *b* be the number of transmissions of *M*_1_ from heterozygous parents to the affected offspring, and let *c* be the number of such transmissions of *M*_*2*_. We then obtain $\hat{\beta }=\,\mathrm{log}(b/c)$ and $\hat{V}=\frac{b+c}{bc}$. The prior distribution of the true effect sizes is assumed to be a normal distribution, i.e., *β*~*N*(0,*W*). Throughout this work, we follow the WTCCC GWAS^6^ to specify the prior variance, i.e., *W* = 0.2^2^ = 0.04. The prior distribution is presented in the left column of Figure S5 of our supplementary information. This method is designated by “ADABF.”

To evaluate the performance sensitivity of ADABF using this prior, we also specify another prior variance, i.e., *W* = 0.1^2^ = 0.01 (right column of Figure S5). This method is designated by “ADABF1.”

According to Wakefield^7,52^, the BF is as follows:

3 $$BF=\sqrt{\frac{\hat{V}}{\hat{V}+W}}\exp (\frac{{\hat{\beta }}^{2}W}{2\hat{V}(\hat{V}+W)}),$$

where ${\hat{\beta }}^{2}/\hat{V}$ is the Wald statistic. For unrelated subjects, $\hat{\beta }$ and $\hat{V}$ are the MLE and its corresponding variance from the linear regression (continuous traits) or logistic regression (dichotomous traits) for a particular variant. The greater the significance of an association, the larger the ${\hat{\beta }}^{2}/\hat{V}$ and BF. Given a fixed $\hat{\beta }=\,\mathrm{log}(1.2)=0.18$, a larger $\hat{V}$ corresponds to a lower power and a decrease in the BF, because the power is not sufficient for providing strong evidence supporting *H*_1_ (Figure S6). Given a fixed *P*-value = 0.02 (i.e., a fixed ${\hat{\beta }}^{2}/\hat{V}$), the BF is small when $\hat{V}$ is extremely small (Figure S7). An extremely small $\hat{V}$ implies an extremely small ${\hat{\beta }}^{2}$ given a *P*-value of 0.02, and thus, the data are unlikely under *H*_1_. Moreover, a large $\hat{V}$ represents a low power that is not sufficient for supporting *H*_*1*_ (Figure S7).

### Ranking by Bayes factor *vs*. *P*-value

**Figure 4:**
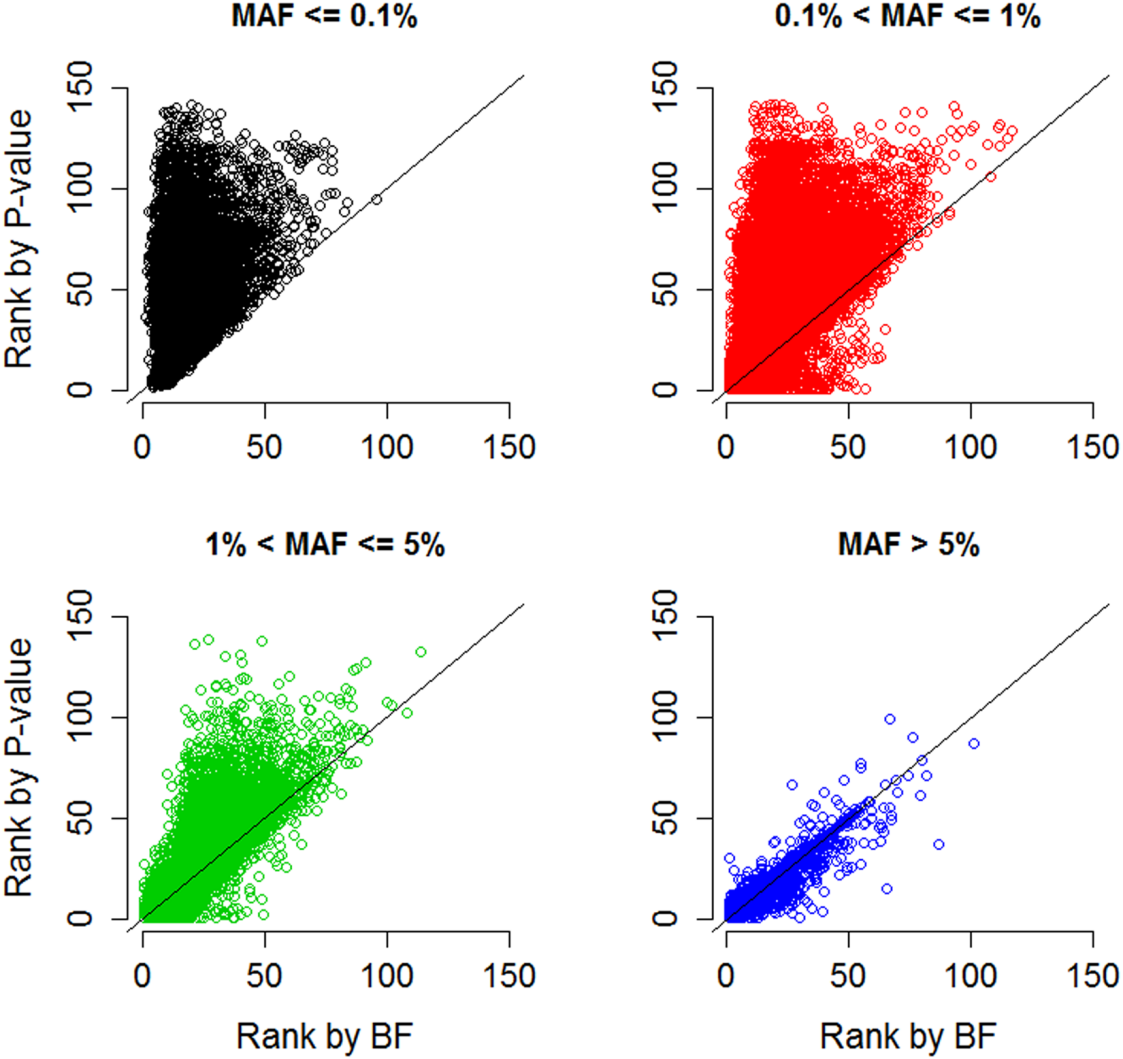
Ranking by Bayes factor *vs*. *P*-value. We performed 200,000 simulations to compare the rankings of a causal variant using the Bayes factor (*x*-axis) and the *P*-value (*y*-axis). The chromosomal region included ~150 rare or common variants, and one of these variants was specified as the causal variant. The scatter plot was stratified according to the MAF of the causal variant. The black line in each plot represents *x* = *y*.

In this subsection, we show that the BF ranking is superior to the *P*-value ranking, in a region with a mixture of rare and common variants. We performed 200,000 simulation replications to compare the rankings of a causal variant by the BF or *P*-value. In each replication, one of ~150 variants was specified as the causal variant. As described in the simulation study, a mixture of rare and common variants was observed in the region (see Figure S8). The disease status (*Y* = 1 denotes disease) was generated according to the following model:

4 $${logit}\,{P}(Y=1)=\alpha +\beta {G}^{c},$$

where $\alpha =\,\mathrm{log}(0.05/0.95)=-2.94$, implying a disease prevalence of 5%. *G* ^*c*^ was the genotype score (0, 1, or 2) of the causal variant, and the effect size was $\beta =\,\mathrm{log}(1.5)$. In total, 200,000 replicates were performed to compare the ranking of a causal variant by the BF (*x*-axis in Fig. 4) to that by the *P*-value (*y*-axis in Fig. 4). The results shown in Fig. 4 and Table 6 are stratified according to the MAF of the causal variant.

**Table 6:** Ranking of a causal variant (a smaller rank is better) in 200,000 replications.

**MAF of the causal variant**	**MAF** <= **0**.**1**%	**0**.**1**% < **MAF** <= **1**%	**1**% < **MAF** <= **5**%	**MAF** > **5**%
Mean rank of the causal variant by BF	17.9	16.6	10.3	4.1
Mean rank of the causal variant by *P*-value	50.3	29.8	12.0	4.1
# (replications where BF ranking was superior)*	46,027	33,271	13,665	1,551
# (replications where BF ranking was identical to *P*-value ranking)*	773	4,747	25,832	47,160
# (replications where BF ranking was inferior)*	3,200	11,982	10,503	1,289
Total	**50**,**000**	**50**,**000**	**50**,**000**	**50**,**000**

^*^Let *R*_*B*_ and *R*_*P*_ be the ranking of the causal variant by the BF and *P*-value, respectively. The following three outcomes could be obtained: (1) the BF ranking was superior if *R*_*B*_ < *R*_*P*_; (2) the BF ranking was identical to the *P*-value ranking if *R*_*B*_ = *R*_*P*_; and (3) the BF ranking was inferior if *R*_*B*_ > *R*_*P*_.

Let *R*_*B*_ and *R*_*P*_ be the rankings of the causal variant by BF and *P*-value, respectively. For a region containing 150 variants, 1 ≤ *R*_*B*_, *R*_*P*_ ≤ 150. A smaller rank would be better, meaning that the causal variant would be ranked in priority order. The following three outcomes could be obtained: (1) the BF ranking was superior if *R*_*B*_ < *R*_*P*_, (2) the BF ranking was identical to the *P*-value ranking if *R*_*B*_ = *R*_*P*_, and (3) the BF ranking was inferior if *R*_*B*_ > *R*_*P*_. According to Table 6, the mean rank of the causal variant by the BF was smaller than (or equal to) that by the *P*-value, across all ranges of causal-allele frequencies. As the MAF of the causal variant increased, the power to detect that causal variant also increased and both mean ranks improved. More replicates showed that the BF ranking outperformed the *P*-value ranking, across all ranges of causal-allele frequencies.

Compared with the *P*-value ranking, rare causal variants will benefit from the BF ranking (see the top-left plot of Fig. 4). This finding can be attributed to a rare causal variant generally having a larger *P*-value (say, *P*-value = 0.2) and a larger $\hat{V}$ (say, $\hat{V}$ = 0.1). As shown in Figure S9(c), its BF will be larger than that of a common neutral variant with the same *P*-value but a smaller $\hat{V}$ (say, $\hat{V}$ = 0.005). That is, a common variant with a *P*-value = 0.2 may actually be a neutral variant, because this large *P*-value is obtained from reliable information (smaller $\hat{V}$). However, a rare variant with a *P*-value = 0.2 may still be causal, because this large *P*-value is obtained from less reliable information (larger $\hat{V}$). Rare variants seldom have small *P*-values, and therefore, our previous ADA method^2^ prioritizes the rare variants with *P*-values smaller than 0.2. However, in a region with a mixture of rare and common variants, a *P*-value threshold of 0.2 is too liberal for common variants. In this situation, it will be better to consider the “relative” evidence in favor of *H*_1_ (i.e., BF), instead of *P*-values.

### Summarizing the BFs in a Chromosome Region

Let *BF*_*l*_ be the BF of the *l* ^th^ variant. Denote the ordered BFs by *BF*_(1)_ ≤ *BF*_(2)_ ≤ … ≤ *BF*_(*L*)_ for a region containing *L* variants. The summary score aggregating the highest *k* BFs is as follows:

5 $${S}_{k}=\sum _{l=1}^{L}I(B{F}_{l}\ge B{F}_{(k)})\mathrm{log}(B{F}_{l}),\,k=1,\cdots ,L,$$

where *I*(*BF*_*l*_ ≥ *BF*_(*k*)_) is 1 if the *l* ^th^ variant is among the top *k* most significant variants according to BF, and is 0 otherwise. Because the natural logarithm of the BF is linked to log-likelihoods, log(*BF*) is considered the “weight of evidence”^10^. Therefore, in Eq. (5), we summarize the association evidence provided by *L* variants in the region of interest. Because log(*BF*) represents the “weight of evidence”^10^, we do not impose any additional weight according to the MAF. As previously mentioned, the MAF has little relevance to association signals^14,15^. Disease-associated variants can be either rare or common. If we believe that rare variants are more likely to be non-neutral, the *Beta*(*MAF*;1,25) function can be used to weight the contribution of individual BFs.

Based on Eq. (5), we obtain *S*_1_, …, *S*_*L*_ for a region containing *L* variants. Then, we use the efficient sequential resampling approach proposed by Liu *et al*.^26^ to assess the significance of the association between the region and a disease. The procedure is performed as follows:

(i) We first draw *B* = 100 sets of ${\hat{{\beta }}}_{0}$ (the *L* × 1 vector of point estimates under the null hypothesis) from the multivariate normal distribution *N*(**0**_*L*×1_,***V***_*L*×*L*_), where the (*i*, *j*)^th^ element of ***V***_*L*_ × _*L*_ is ${R}_{i,j}\sqrt{{\hat{V}}_{i}{\hat{V}}_{j}}$. ${\hat{V}}_{i}$ and ${\hat{V}}_{j}$ are the estimated variances of ${\hat{\beta }}_{i}$ and ${\hat{\beta }}_{j}$, respectively [*i*, *j* = 1, …, *L*. Recall that ${\hat{\beta }}_{i}$ and ${\hat{V}}_{i}$ are obtained from a regression model of the *i* ^th^ variant, such as Eq. (2)]. Yang *et al*.^53^ have shown that the correlation among the association statistics in a region can be well approximated by the correlation among the genotypes. Therefore, *R*_*i*,*j*_ is estimated from the correlation of the genotypes at the *i* ^th^ and *j* ^th^ loci. When analyzing case-parent trios, only the founder genotypes are used to calculate *R*_*i*,*j*_.
(ii) For the *b* ^th^ set of ${\hat{{\boldsymbol{\beta }}}}_{0}$, we calculate the BFs using Eq. (3) and the summary scores using Eq. (5). Given *k* (*k* = 1, …, *L*), we compare *S*_*k*_ with *S*_*k*_^(*b*)^ (*b* = 1, …, *B*) and obtain the *P*-value of *S*_*k*_ by $[{\sum }_{b=1}^{B}I({S}_{k}^{(b)}\ge {S}_{k})]/B$. In the observed sample, we find the minimum *P*-value across *k* (*k* = 1, …, *L*), which is denoted by *MinP*. The minimum *P*-value of the *b* ^th^ resample is calculated similarly and denoted by *MinP* ^(*b*)^, *b* = 1, …, *B*. Finally, the adjusted *P*-value is $[{\sum }_{b=1}^{B}I(Min{P}^{(b)}\le MinP)]/B$.
(iii) If the adjusted *P*-value based on 100 sets of ${\hat{{\boldsymbol{\beta }}}}_{0}$ is smaller than 0.1, we draw 10 times more sets (i.e., *B* = 1,000) to increase the precision of the *P*-value. This procedure is repeated until the *P*-value is larger than $10/B$ or a desired precision level is reached.

The R code of our ADABF method can be downloaded from http://homepage.ntu.edu.tw/~linwy/ADABF.html.

### Competitor Methods

The ADABF test (prior variance *W* = 0.2^2^ = 0.04) was compared with the ADABF1 (prior variance *W* = 0.1^2^ = 0.01) and ADA tests^2^. To make a fair comparison, these three tests were all performed using the “adaptive rank truncated product” (ARTP) method^11,12^. Therefore, the highest *k* BFs (or the smallest *k P*-values) are combined, in the observed sample and in each of the resamples, respectively. The abovementioned sequential resampling approach^26^ was used to assess the significance of the association between the region of interest and the disease, and the minimum and maximum numbers of resampling were set as 10^2^ and 10^7^, respectively.

Furthermore, the TLC and TK tests were performed for comparison. These two tests were performed using the “rvTDT” R package (version 1.0)^16^ and the “SKAT” R package (version 1.2.1)^4,19,28^, to analyze the case-parent trios and unrelated subjects, respectively. To make a fair comparison, we did not assign any MAF-weighting function to the TLC, TK, ADABF, ADABF1, or ADA tests.

For the analysis of unrelated subjects, we also compared ADABF with VW-TOW^18^. VW-TOW is a test used to detect associations of rare and common variants, and was proposed by Sha *et al*.^18^. These authors divided the variants into rare (if MAF < RVT) and common (if MAF ≥ RVT), and then searched for the optimal weights for the two groups of variants, separately. The statistics from the two parts of the variants were then combined, and the *P*-value was calculated with permutations. This test was performed using the R code, which was downloaded from the authors’ website at http://www.math.mtu.edu/~shuzhang/software.html, and the number of permutations was set as 10,000. The RVT was set as $1/\sqrt{2n}$, where *n* was the sample size^4^. Because VW-TOW was proposed for the analyses of unrelated individuals^18^, it was not evaluated for case-parent trio data.

### Simulation Study

With the Cosi program^54^, we generated 100 data sets following the LD patterns in Europeans. Each of the 100 Cosi data sets contained 10,000 chromosomes from a 20 kilo base (kb) pairs region. That is, totally 100 20-kb regions were considered. On average, ~150 variants could be observed in a 20-kb region. The distribution of the MAFs of the variants is shown in Figure S8 in the supplementary information, which presents as an L-shaped distribution that is typical of allele frequencies^55^.

To evaluate the type I error rates, the disease status (*Y* = 1 denotes disease) was generated according to the following model:

6 $$logit\,P(Y=1)=\alpha ,$$

where *α* = −2.94. To study the power, the disease status was generated according to the following model:

7 $$logit\,P(Y=1)=\alpha +{\beta }_{1}{G}_{1}^{c}+\cdots +{\beta }_{d}{G}_{d}^{c},$$

where *α* = −2.94, *d* was the number of causal variants (*d* = 2 or 4), *G*_*k*_^*c*^ was the genotype score (0, 1, or 2) of the *k* ^th^ causal variant, and the effect sizes were $\beta \text{'}s=\pm \mathrm{log}(1.5)$ or $\pm \mathrm{log}(1.25)$. *β* was positive or negative depending on whether the causal variant was deleterious or protective, respectively. When $\beta =\pm \mathrm{log}(1.5)$, the odds ratio (OR) was 1.5 for a deleterious allele and $1/1.5=0.67$ for a protective allele. When $\beta =\pm \mathrm{log}(1.25)$, the OR was 1.25 for a deleterious allele and $1/1.25=0.8$ for a protective allele. The following two scenarios were evaluated:

1. All causal variants were deleterious and
2. In total, 50% of the causal variants were deleterious, and 50% of the causal variants were protective.

The following two data structures were simulated: (1) 2,000 case-parent trios and (2) 2,000 unrelated subjects, of which 1,000 were cases, and 1,000 were controls.

## Electronic supplementary material

Supplementary Information
