# Supplementary material for: Adaptive combination of Bayes factors as a powerful method for the joint analysis of rare and common variants
Source: Sci Rep. 2017 Oct 24;7:13858. doi: 10.1038/s41598-017-13177-7 (PMC5654754; doi:10.1038/s41598-017-13177-7)
Supplement: Supplementary file 1 — Supplementary Information [file 41598_2017_13177_MOESM1_ESM.pdf]

Adaptive combination of Bayes factors as a powerful method for the joint analysis of rare and common variants

Wan-Yu Lin [1,2]*, Wei J. Chen [1,2,3,4], Chih-Min Liu [5], Hai-Gwo Hwu [1,5], Steven A. McCarroll [6,7,8], Stephen J. Glatt [9], Ming T. Tsuang [10,11]

[1] Institute of Epidemiology and Preventive Medicine, College of Public Health, National Taiwan University, Taipei, Taiwan
[2] Department of Public Health, College of Public Health, National Taiwan University, Taipei, Taiwan
[3] Institute of Brain and Mind Sciences, College of Medicine, National Taiwan University, Taipei, Taiwan
[4] Genetic Epidemiology Core Laboratory, Division of Genomic Medicine, Research Center for Medical Excellence, National Taiwan University, Taipei, Taiwan
[5] Department of Psychiatry, College of Medicine and National Taiwan University Hospital, National Taiwan University, Taipei, Taiwan
[6] Stanley Center for Psychiatric Research, Broad Institute of MIT and Harvard, Cambridge, MA, USA
[7] Program in Medical and Population Genetics, Broad Institute of MIT and Harvard, Cambridge, MA, USA
[8] Department of Genetics, Harvard Medical School, Boston, MA, USA
[9] Departments of Psychiatry and Behavioral Sciences and Neuroscience and Physiology, Medical Genetics Research Center, SUNY Upstate Medical University, Syracuse, New York, USA
[10] Center for Behavioral Genomics, Department of Psychiatry, University of California San Diego, La Jolla, California, USA
[11] Institute for Genomic Medicine, University of California San Diego, La Jolla, California, USA

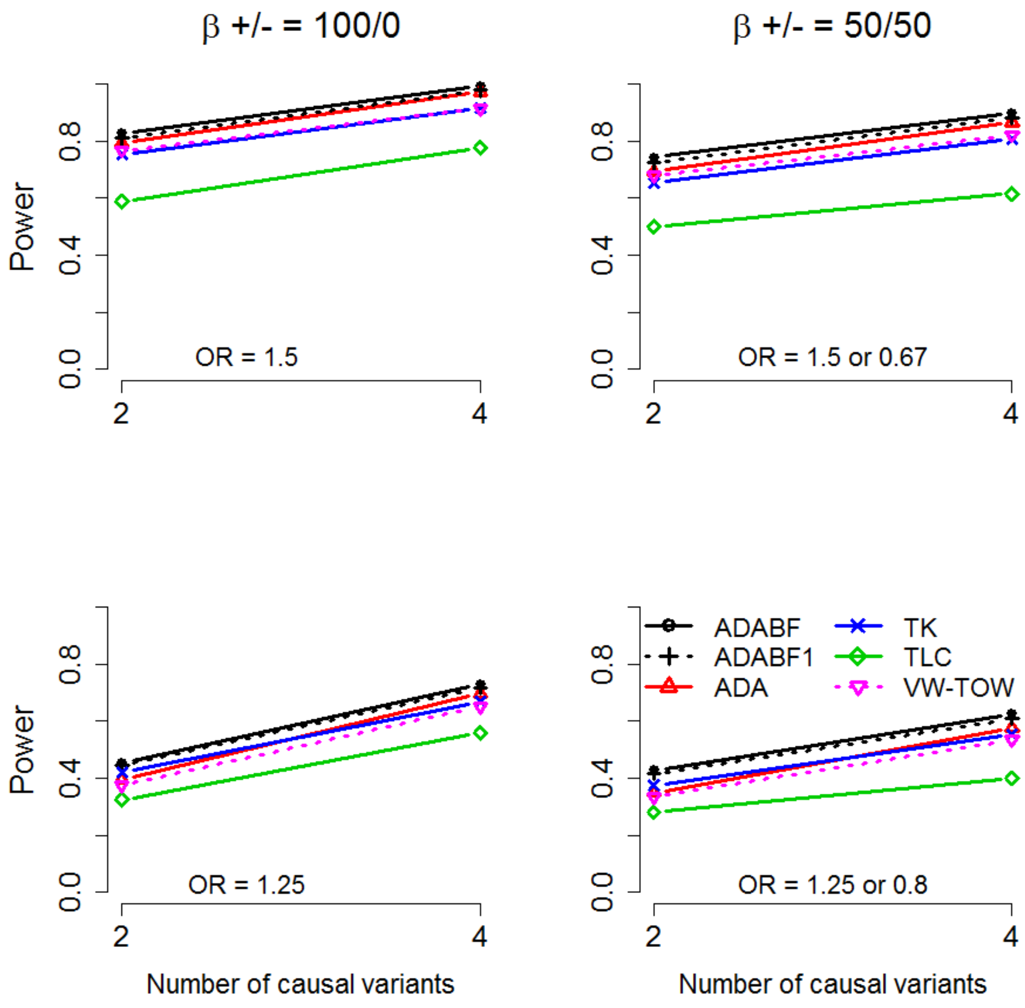

Figure S1. Simulation results of the unrelated cases and controls (haplotypes generated according to the LD patterns in Europeans) (given the significance level of 0.01)

Top row: OR = 1.5 for a deleterious allele and OR = 0.67 for a protective allele; bottom row: OR = 1.25 for a deleterious allele and OR = 0.8 for a protective allele. Left column: all causal variants were deleterious; right column: ~50% of the causal variants were deleterious, and the other ~50% were protective. The x-axis shows the number of causal variants, whereas the y-axis shows the power.

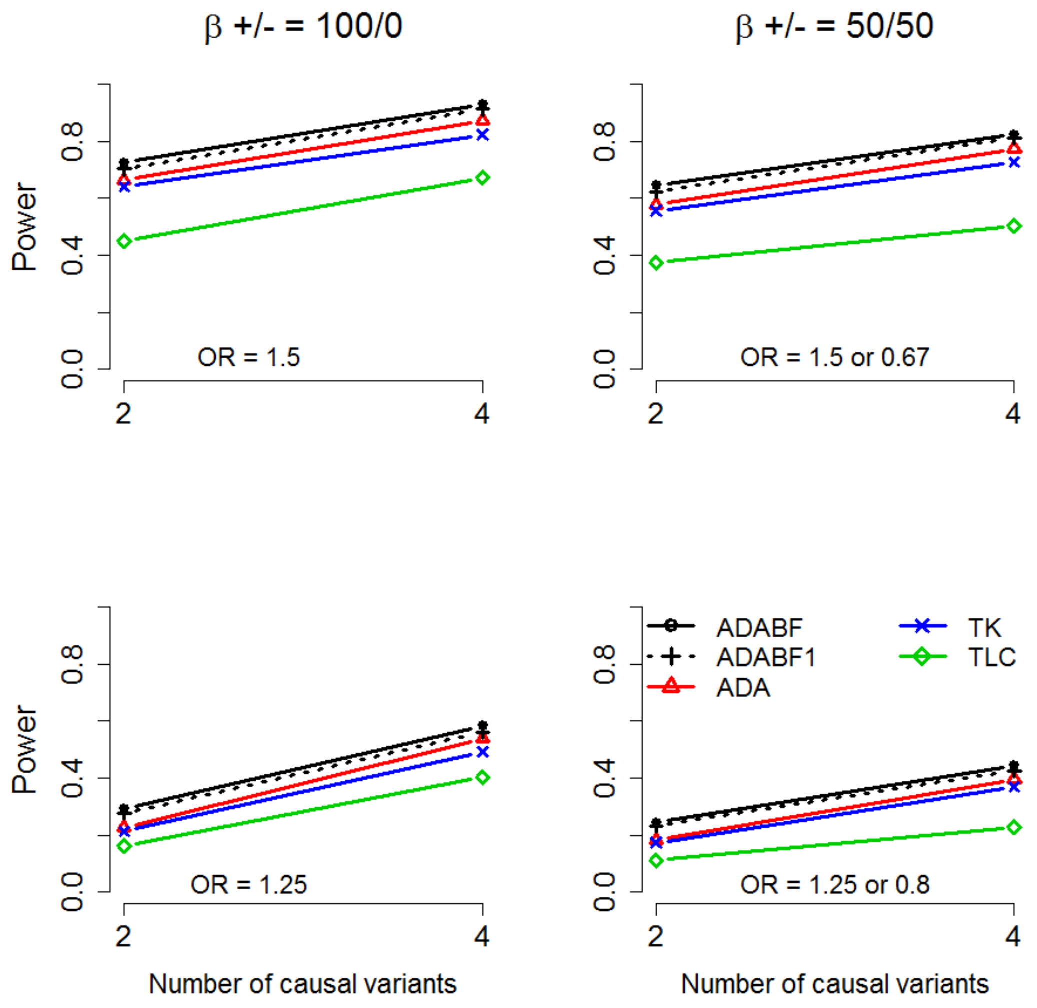

Figure S2. Simulation results of the case-parent trios (with Asian linkage disequilibrium patterns) (given a significance level of 2.5×10^{-6})

Top row: OR = 1.5 for a deleterious allele and OR = 0.67 for a protective allele; bottom row: OR = 1.25 for a deleterious allele and OR = 0.8 for a protective allele. Left column: all causal variants were deleterious; right column: ~50% of the causal variants were deleterious, and the other ~50% were protective. The x-axis shows the number of causal variants, whereas the y-axis shows the power.

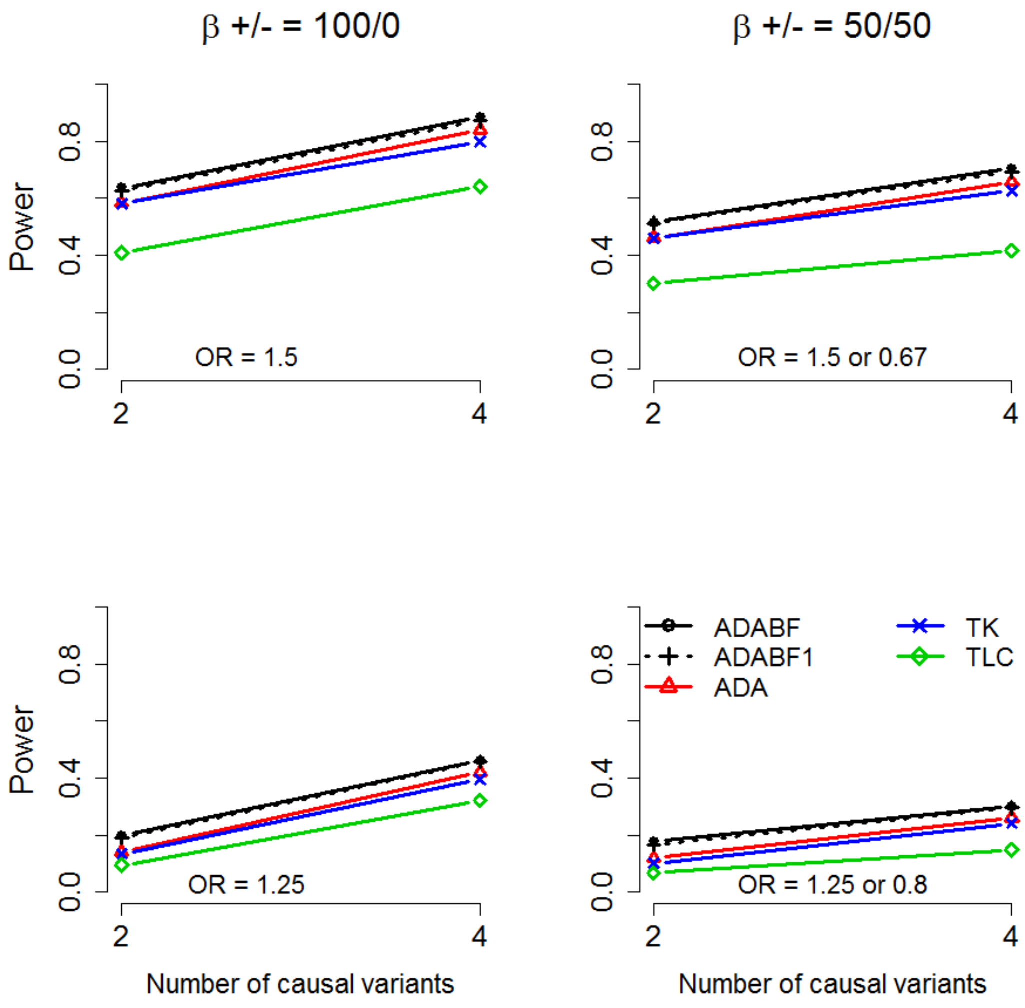

Figure S3. Simulation results of the unrelated cases and controls (with Asian linkage disequilibrium patterns) (given a significance level of 2.5×10^{-6})

Top row: OR = 1.5 for a deleterious allele and OR = 0.67 for a protective allele; bottom row: OR = 1.25 for a deleterious allele and OR = 0.8 for a protective allele. Left column: all causal variants were deleterious; right column: ~50% of the causal variants were deleterious, and the other ~50% were protective. The x-axis shows the number of causal variants, whereas the y-axis shows the power.

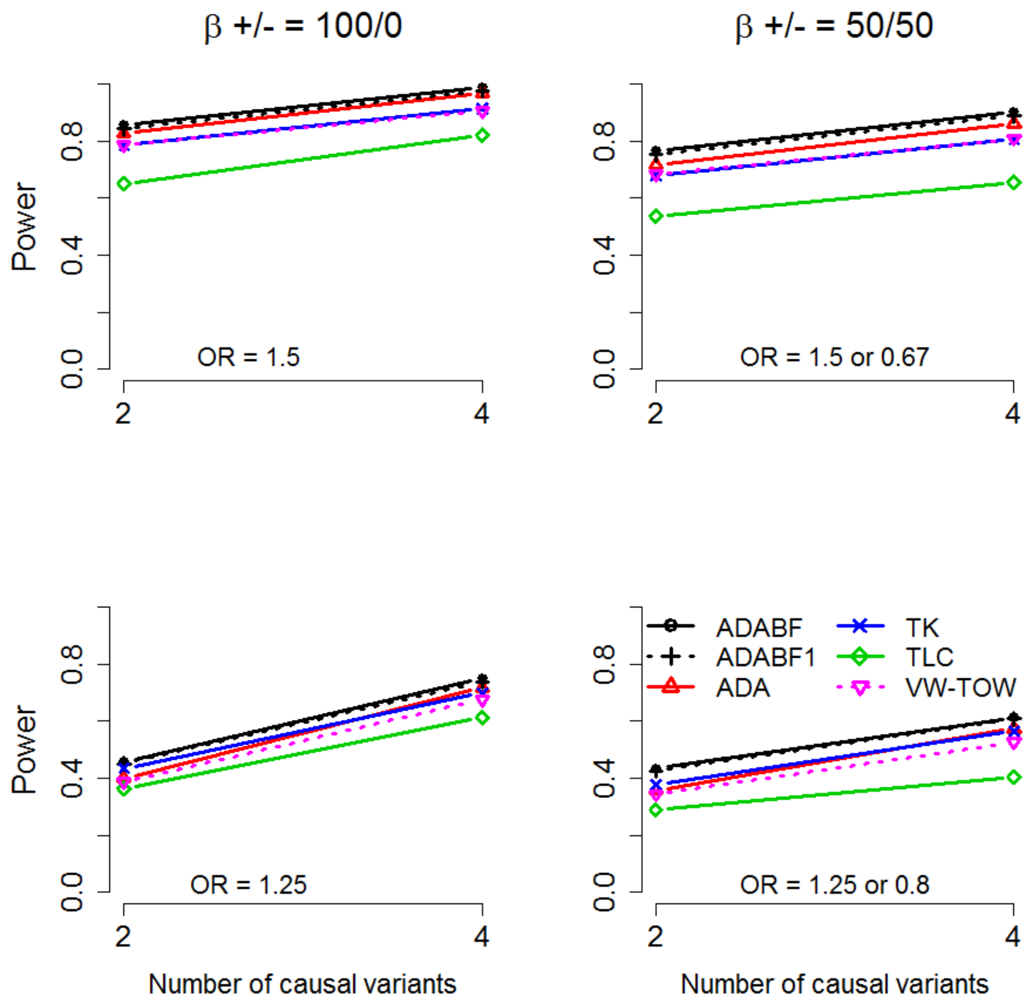

Figure S4. Simulation results of the unrelated cases and controls (with Asian linkage disequilibrium patterns) (given a significance level of 0.01)

Top row: OR = 1.5 for a deleterious allele and OR = 0.67 for a protective allele; bottom row: OR = 1.25 for a deleterious allele and OR = 0.8 for a protective allele. Left column: all causal variants were deleterious; right column: ~50% of the causal variants were deleterious, and the other ~50% were protective. The x-axis shows the number of causal variants, whereas the y-axis shows the power.

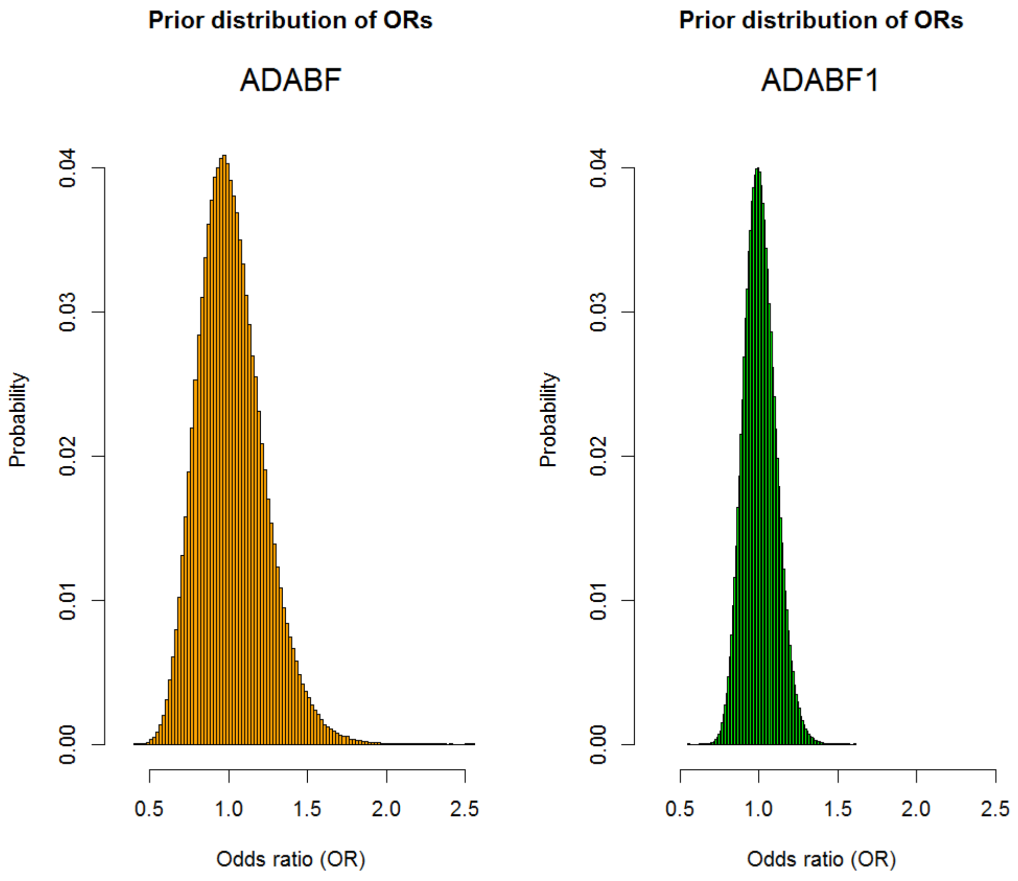

Figure S5. The prior distribution of odds ratios (ORs) used in ADABF and ADABF1, respectively

The prior for ADABF (left) is the prior used by the WTCCC genome-wide association study (WTCCC, 2007).

[References]

Wtccc (2007) Genome-wide association study of 14,000 cases of seven common diseases and 3,000 shared controls. *Nature,* 447**,** 661-78.

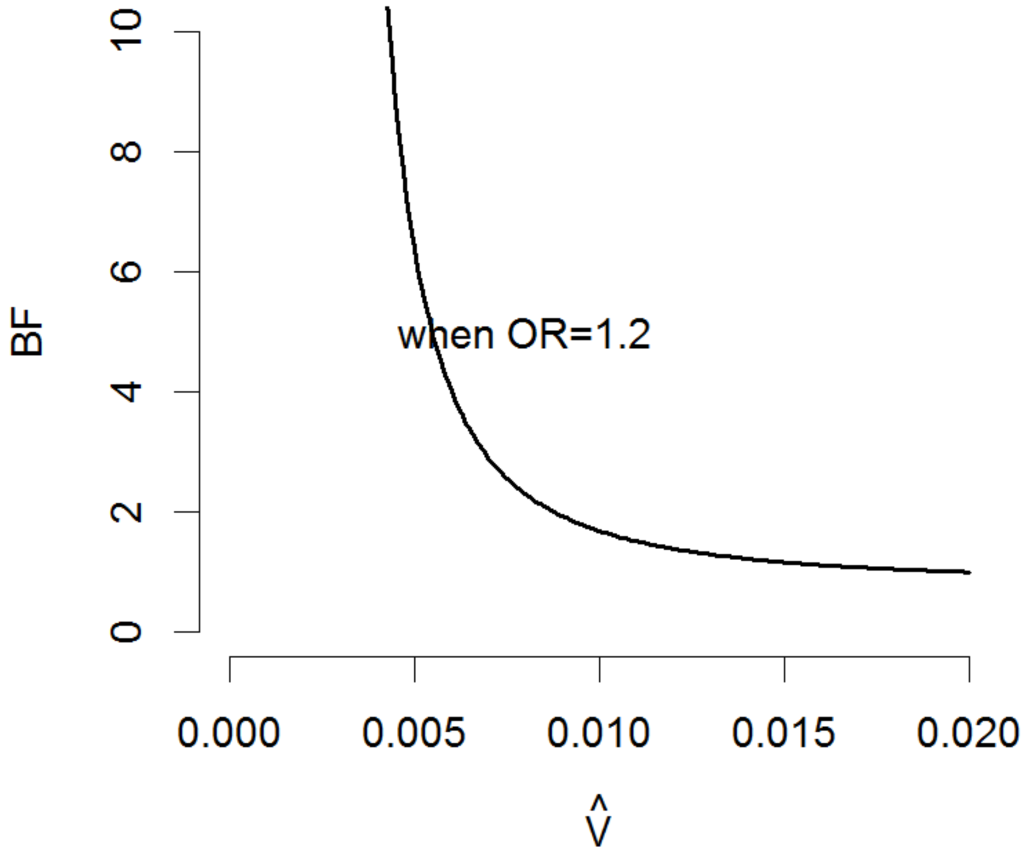

Figure S6. Bayes factor (BF), or the evidence in favor of the alternative hypothesis (H_1), as a function of the variance estimate $\hat{V}$, for a fixed OR = 1.2 and W = 0.04 (following the WTCCC 2007 paper). A larger $\hat{V}$ corresponds to a lower power and a decrease in the BF, because the power is not sufficient to provide strong evidence supporting H_1.

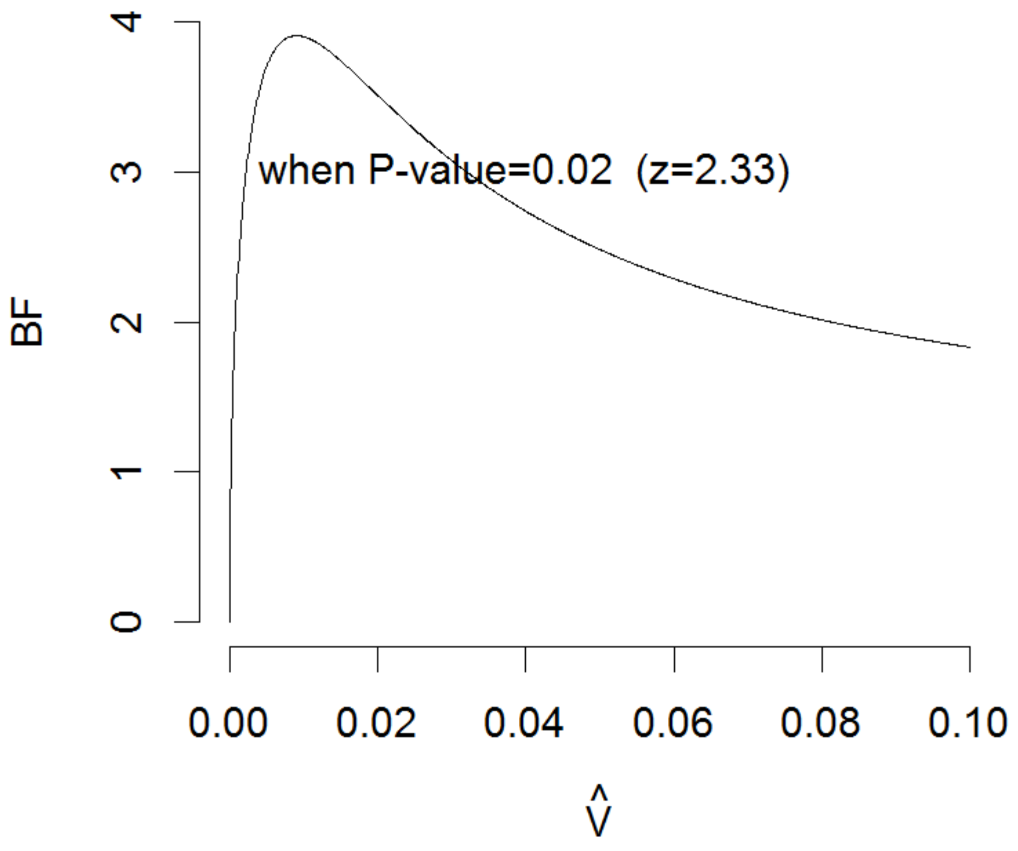

Figure S7. Bayes factor (BF), or the evidence in favor of the alternative hypothesis (H_1), as a function of the variance estimate $\hat{V}$, for a fixed P-value = 0.02 (i.e., $z = \hat{\beta}/\sqrt{\hat{V}}$ = 2.33) and W = 0.04 (following the WTCCC 2007 paper).

(1) Given a fixed P-value (i.e., a fixed $\hat{\beta}/\sqrt{\hat{V}}$), the BF is small when $\hat{V}$ is extremely small. This is because an extremely small $\hat{V}$ implies an extremely small $\hat{\beta}$, and the data are unlikely under H_1.

(2) When $\hat{V} > W/(z^2-1) = 0.04/(2.33^2-1) = 0.009$, the BF is decreasing because the power is not sufficient to provide strong evidence in favor of H_1.

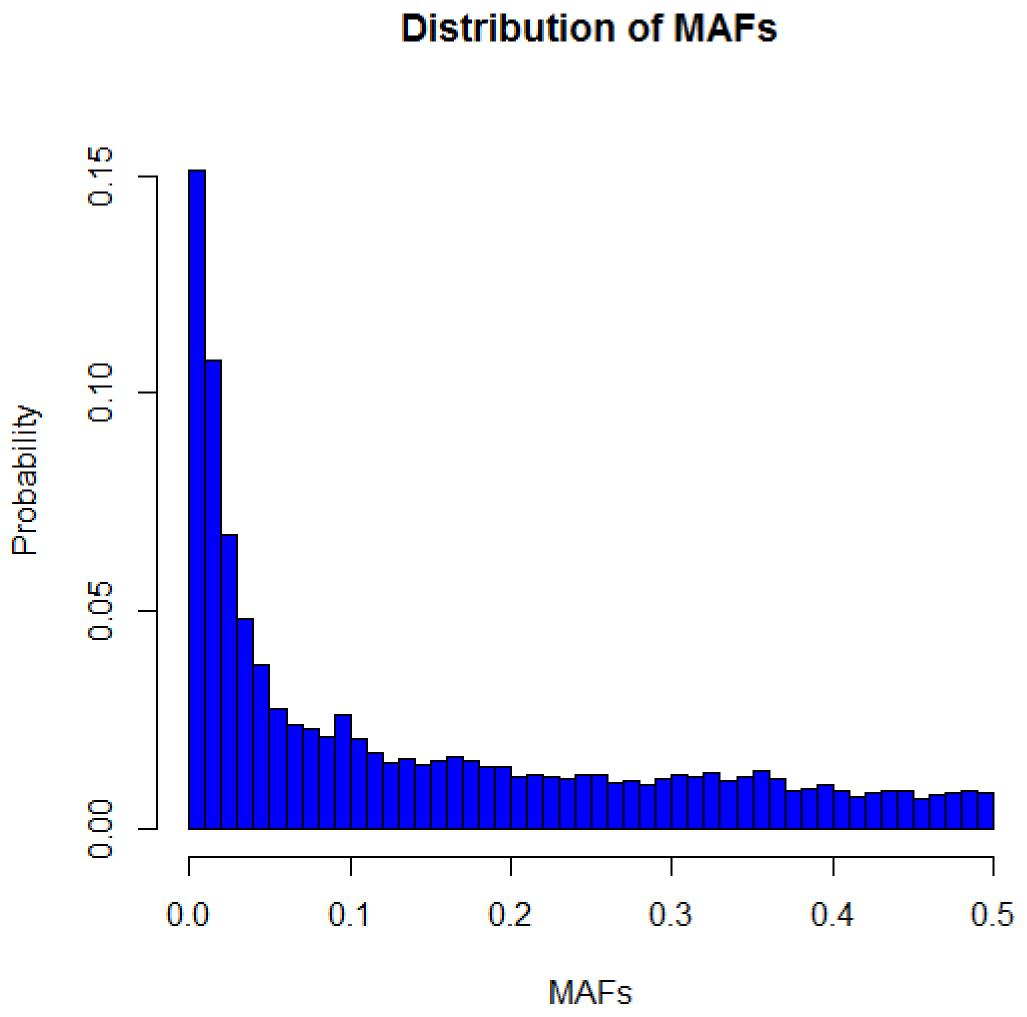

Figure S8. The typical L-shaped distribution of minor allele frequencies (MAFs) of variants simulated from the Cosi program (the invariant loci with MAFs of 0 have been removed)

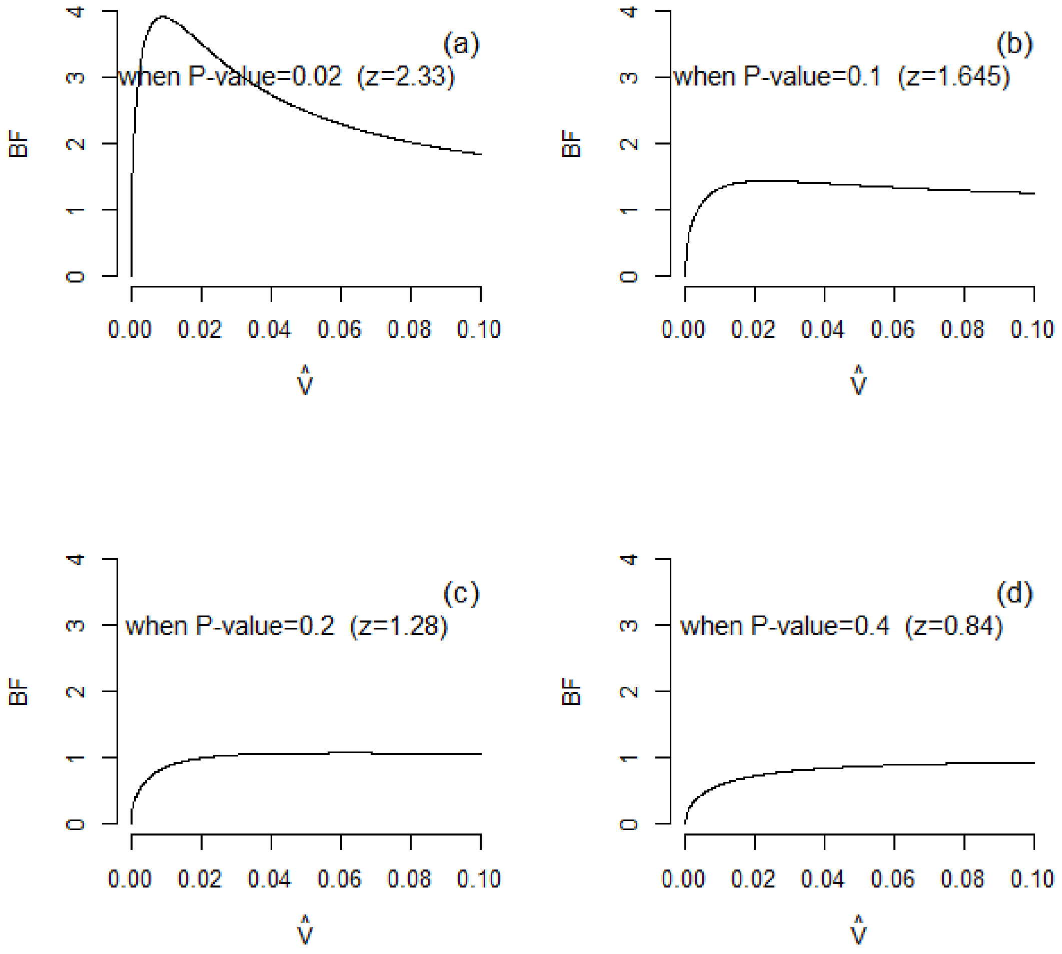

Figure S9. Bayes factor (BF), or the evidence in favor of the alternative hypothesis (H_1), as a function of the variance estimate $\hat{V}$, for a fixed *P*-value = 0.02, 0.1, 0.2, and 0.4; *W*=0.04 (following the WTCCC 2007 paper).
